# Pipped at the Post: Knowledge Gaps and Expected Low Parental IT Competence Ratings Affect Young Women’s Awakening Interest in Professional Careers in Information Science

**DOI:** 10.3389/fpsyg.2019.00968

**Published:** 2019-05-01

**Authors:** Angela Schorr

**Affiliations:** Media Psychology Lab, Institute of Psychology, Faculty II, University of Siegen, Siegen, Germany

**Keywords:** computer competence, computer affinity, gender stereotypes, ICT socialization, ICT professions, robotics, attitude change, vocational counseling

## Abstract

Although many jobs in today’s information science allow favorable work-life-schedules for women, they still hesitate to enter this territory. In a study based on individual interviews with *N* = 134 students aged 14–18 years, who visited the *Deutsches Museum* in Munich, Germany, we collected data on the students’ socialization in information and communication technology (ICT), on their self-rated ICT competence, their working knowledge of ICT professions, and their reaction to sexist statements. To analyze more in depth, we provided the participants with two alternative forms of vocational counseling interventions designed to modify their ICT-related attitudes (information vs. robotics condition). Analyses of variance and multiple linear regressions were administered to the data. Results: The girls in this study were socialized more than one year after the boys in using computers. While the boys received their ICT training mostly through their fathers and peers, the girls frequently had to rely on their teachers for ICT instruction. The girls rate their ICT competence lower than the boys; nevertheless, both genders share a relatively high interest in ICT professions. What’s more, the girls are less convinced that men have a natural talent for computer science. Openness toward taking up jobs in the ICT industry in the case of the boys is less determined by their self-rated computer competence and the perceived ICT talent assessment by their parents. In both intervention conditions, they eagerly received and processed the new information provided. The girls’ interest in an ICT career largely depends on preconditions, namely on their self-rated ICT competence, on a long-standing enthusiasm for computers, and on what they perceive their parents think about their ICT talents. Unlike the more pragmatic approach of the boys, their self-doubts, especially among the academic high school girls brings about that they are still in danger to leave the field of information/computer science before having entered it. In general, the participants’ responses point to a comprehensive misdirection of young women in German middle schools and academic high schools. Fortunately, this study provides a lot of evidence on how to fix this major mishap in the interest of both sexes.

## Introduction

How is it, that so few young women are choosing information and communication technology (ICT) careers? Graduates of professions in the ICT sector (either with a middle school/MS diploma plus a vocational training in information technology or with an academic high school/AHS diploma plus a university degree in computer science) are very sought after. The job prospects are excellent. Training in media professions is in vogue, while the computer science economy in Germany has recruitment problems, which cannot be explained solely by the growing demand for IT services. Although many fields of work in the ICT area are characterized by high space-time independence and flexibility and thus can be classified as family friendly, young women in particular seldom opt for these careers.

### Young Women in Computer Science: Self-Doubts and Ambivalent Feelings of Belonging

Already at the turn of the millennium German experts were able to identify potentially important factors based on representatively collected survey data, which could explain the small proportion of women in computer science studies – at that time their share was 7–8% (Schinzel et al., 1999; Ihsen et al., 2013). It turned out that the parents and the school seemed to inhibit the young women to become interested in computer science. “Accordingly, women often decide to study computer science only after graduation…” (Schinzel et al., 1999, p. 13). These ICT-inclined young women started their course of studies with less confidence, especially in terms of computer knowledge, programming and software. More often than their male counterparts, they said, they felt they were inferior despite good performances. Less often, as was the case with male classmates hobbies and work overlapped. Overall, the authors concluded: “The women are much more doubtful about their abilities and suitability for the subject. The conversations among the students unsettle them, especially in the early phase of their studies” (Schinzel et al., 1999, p. 13, translated by the author). These findings are consistent with studies in other countries and other STEM fields. Despite equally good performances, in computer science as well as in other STEM fields like e.g., engineering young women regularly show lower competence perceptions compared to young men (Reid, 2009; Jagacinski, 2013). That these research results continue to be relevant today is shown by a study recently published by Ito and McPherson (2018). The authors surveyed 386 high school students and their results add another piece to the big puzzle: For female students but not for male students, they could show that the self-rated likelihood of persisting in the current STEM class, to pursue the topic further in high school and college, and to pursue a career in STEM can be significantly predicted by the social belonging dimension, here defined as feeling of acceptance and fit with the STEM class.

It remained unclear how exactly the socialization processes in family, school, vocational training and studies run their course, keeping young women in Germany away from the computer science professions (Ebach, 1994; Schinzel and Ruiz Ben, 2004; Maaß and Wiesner, 2006; Ehrke et al., 2011). Two decades of research culminated in compendiums with more than 30 differentiated recommendations for gender-appropriate higher education (Ihsen et al., 2017; Resch et al., 2017). Instead of tackling the root of the problem, experts and stakeholders such as the German industry association *bitkom*, given the shortage of skilled workers, increasingly recommend mono-educational courses and pure women’s study courses (Nordmann, 2016; Bitkom Press, 2018). These are approaches that the female computer science students surveyed at the turn of the millennium already rejected (Schinzel et al., 1999). In 2018, still only 17% of ICT professionals in Germany are women.

### Research on the Impact of Parents, Teachers, and School Curricula

Ihme and Senkbeil (2017) carried out a scientific study with 224 students at middle schools in the 8th grade, half girls and boys. They come to the conclusion that boys rate their computer-related skills significantly higher than girls, even though there are in fact no differences in competencies. For the traditional STEM field of mathematics Gunderson et al. (2012a,b) based on a comprehensive research review conclude that parents’ and teachers’ expectancies for children’s math competence are still often gender biased. The results of a recent representative study by Lloyd et al. (2018) on the informal learning world “family” are pointing in the same direction. They looked at 6,492 Australian children aged 3–12 and came to the sobering conclusion, “Even when parents created a supportive environment, there was little evidence indicating that girls were encouraged to pursue STEM” (Lloyd et al., 2018, p. 308). In addition to the teachers, these days especially the fathers are targeted by researchers. In the study Galdi et al. (2017) published on the family and school environment in 68 six-year-old children, they could prove that the fathers’, but not the mothers’, stereotypical beliefs predicted children’s stereotypes.

As far as German students are concerned, Ihme and Senkbeil (2017) mainly blame the formal learning world “school.” They found that the shortcomings of school education have serious consequences for girls and boys alike: Due to the missing curricular implementation of computer-related skills in Germany and the resulting lack of feedback, adolescents tend to overestimate their abilities in the area of computer-related skills. As a result, their positive self-assessment depends on the frequency of computer use and leads to a skills illusion. German schools hardly have a chance to compensate for social disparities in the acquisition of computer-related skills. In everyday life, according to the authors, the adolescents acquire these skills mainly through the help of their parents.

### Research on the Impact of Gender Stereotypes, Stereotype Threat, and Domain Masculinity

Part of the current research on gender issues in the STEM domains is dedicated to researching the impact of gender stereotypes, stereotypical threat and domain masculinity on girls and young women. Both, the internalization of inferiority images and their consequences (gender stereotypes) as well as the reactions on the immediate situational threat that derives from negative stereotypes disseminated of one’s group (stereotype threat) have an effect on young women in the STEM field. They display cognitive, physiological and performance-related responses like increased stress, lack of self-esteem, increased self-doubt, failure in performance, etc., (Murphy et al., 2010; Régner et al., 2016). This is particularly true of domains in which women are already negatively stereotyped, respectively, domains that are stereotyped as masculine (Schmader et al., 2004; Thoman et al., 2008; Cheryan et al., 2009; Logel et al., 2009;Cheryan, 2012; Hergatt Huffman et al., 2013; Ihsen, 2013).

Today gender parity in school or academic achievements in STEM fields, meaning that girls today are performing just as well or even better as boys in these fields, has been achieved (e.g., Kim et al., 2014). Nevertheless, women continue to choose STEM-related careers at significantly lower rates than men do (Cheryan, 2012; Hergatt Huffman et al., 2013; Régner et al., 2016). Hergatt Huffman et al. (2013) can prove in their study that not only the biological sex plays the decisive role, but rather gender roles, specifically masculinity. Cheryan (2012) concludes “that considering the extent to which math-related domains are stereotyped as masculine can help explain why women do not seek out math-related careers, even as they perform just as well in math” (Cheryan, 2012, p. 184).

Another recent finding on gender interaction in computer science studies confirms that too little progress is being made. Förtsch et al. (2018) in the *Bamberg Alumnae Tracking Study* asked the question: “Do male and female graduates differ in their level of academic achievement in computer sciences? And if not, does ICT change anything?” They found that although the academic achievements of female graduates are as good as those of male graduates, still “female graduates exhibit lower self-belief in their professional skills, partly because lower-achieving male graduates still display very high professional skills self-efficacy beliefs, irrespective of their previous academic achievements at university. (…) The career ambitions and career opportunities of male graduates depend less on their academic achievements at university, whereas female graduates have to be very ambitious to be able to hold a leadership position in the same field” (Förtsch et al., 2018, p. 265).

### Can the Interest in ICT Counteract the Masculine Image of Computer Science? Research on Attitude Change

However, there is also evidence from research that something is changing in the generation of students studied, that both genders approach the ICT domain on their own and because of intrinsic motivation: Sáinz et al. (2016) investigated young people’s gender stereotypes and attitudes about people working in the field of ICT. 900 pupils on average 15 years old were interviewed. Both boys and girls held stereotypical beliefs about ICT as a highly male-dominated field. “As expected, these stereotypical beliefs described a masculine portrayal of ICT workers. Contrary to the expectations, most of the students’ portrayals of people working in ICT were either positive or neutral, not negative” (Sáinz et al., 2016, p. 154). No gender differences were observed in the type of characteristics associated with ICT professionals. Another indication of how young women are actively trying to approach the ICT domain lies in the observation of Sáinz et al. (2016), that “young females were more likely to offer feminine references about the professions where ICT is the tool rather than the object of their work” (Sáinz et al., 2016, p. 154). A similar observation is made by Lasen (2010) in focus group interviews with pupils in the 11th and 12th grade. Both, the group of ICT course participants (takers) as well as the group of those who did not attend ICT courses (non-takers), expressed an aversion to programming. But the interviews also showed that those who did not attend ICT courses misunderstood the purpose of the courses and interpreted them as geared to programming and other highly technical skills only. In the case of the female takers, it was the creative aspects of information technology systems which had attracted them to the subject and they were in fact enjoying ICTs authentic, problem-based design tasks. Lasen concludes, “Findings indicate that schoolgirls’ participation in ICT pathways may well be promoted through subjects that position and call for students to engage with ICTs as ‘enablers’ in diverse, meaningful and creative human contexts” (Lasen, 2010, p. 1117).

### Why It Is Necessary to Focus on the Topic of Career Choices and Vocational Counseling of Young People Interested in ICT

The extensive digitization of everyday life is changing the attitudes of young people toward ICT professions, but in Germany these changes are only slowly taking place. There is a lack of personal and professional support for young women to become interested in a job in the ICT sector. The well-known deficits in ICT socialization are “repaired” with more or less commitment, instead of tackling the problems at all levels, at all ages. To make progress, one needs to analyze the situation more closely.

Generally, young people between the ages of 14 and 18 for the first time want to find out what they want to do later in their lives. The interest in professional advice is high. Also for this reason, the *Deutsches Museum*, which is a technical museum, is a popular destination for young people of this age group. So far, little research has been done on this important orientation phase for the career decision of young people interested in learning something about the ICT sector.

Previous research focused on psychological dimensions such as academic self-concept, perceived parental support, stereotypical threat, sense of social belonging and others (e.g., Nagy et al., 2010; Heerwegh et al., 2016; Ito and McPherson, 2018). For about 15 years, various research teams attempted to elucidate the complex pattern of biographical, family related, and school-related factors affecting the ICT socialization of girls and young women. A number of elaborate studies guided by different research interests were published. In these studies, either primary school age children were included (Meelissen and Drent, 2008; Vekiri and Chronaki, 2008), or college freshman as well as undergraduate and graduate students (e.g., Verhoeven et al., 2010; Heerwegh et al., 2016; Ertl et al., 2017). A good example of these approaches is an exciting recent study by Ertl et al. (2017). They focus on the effects of gender stereotypes on the self-concept of female students in STEM degree programs with less than 30% females. On the topic of academic self-concept in mathematics, Nagy et al. (2010) published an insightful study, which – by the way of exception – specifically covered the age group of 13 to 18 year olds (see also Miliszewska and Sztendur, 2010).

For this study, we have specifically selected the age group of 14 to 18 year olds. The focus of the investigation is thus on the life phase, in which important decisions for the future profession are made. The focus on the ICT professions and the interest in these professions is at the center. Our central research question in this project was: Which factors have an impact on the career choice of young women and young men in terms of their interest in IT careers (e.g., ICT socialization in family and school, parental support, ICT affinity, computer competence, working knowledge of the ICT professions, gender stereotypes or others)? And: How can we change some of these learned, but “dysfunctional” attitudes toward the ICT sector? – Positive results on attitude changes as regards to the attractiveness and fit of the ICT domain for young women appear at least short-term and intervention-based possible. With regard to the research results on gender stereotypes, it is assumed that these continue to be effective. Starting with the topic of young womens’ ICT socialization, the following hypotheses guided the investigation: (1) The ICT socialization, i.e., the individual media use biography and the self-assessed computer and Internet competence have an influence on young womens’ interest in computer science professions. Especially, the informal learning environment “family” has an impact on young womens’ attitudes toward the ICT field. (2) In Germany, the formal learning environment “school” currently cannot compensate for the deficits in the ICT socialization of girls and young women. (3) Informative and targeted vocational counseling interventions can change the interest in ICT professions. (4) Interest in ICT is still influenced by gender stereotypes.

## Materials and Methods

### Design of the Study

This is an interview study in which vocational counseling interventions are integrated. It follows the tradition of educational intervention research (cf. Hunt and Hunt, 2004; Mansoori-Rostam and Tate, 2017; Shiina et al., 2017; Turetsky and Sanderson, 2017). One focus of the study is on the coverage and analysis of the participants’ complex ICT biography (family/school) in connection to gender stereotypes and the participants’ interest in ICT professions. The other focus is on the two vocational counseling intervention conditions we applied. For years and based on careful analyses of research, intervention measures to eliminate gender gaps in the field of computer science were proposed (e.g., Quaiser-Pohl, 2012; Ihsen et al., 2017). New to this study is that by focusing on the psychological dimension of “Interest in ICT professions,” we investigated in two different intervention conditions intended to effect attitude change.

In the German Science and Technology Museum (*Deutsches Museum*) *N* = 134 students were recruited for the project. The participants were approached by the interviewers at the ICT laboratory of the Technical University of Munich (TUMLab) and motivated to participate in the project. The activities in the TUMLab can be seen from the outside through a fully glazed wall for every visitor. At a first glance it looks like a standard computer lab with 15–20 seats. Under the motto “Science to touch and experience” guided automation courses, programming courses, etc., can be booked by schools and other interested parties. However, our respondents did not take part in such courses. They passed the Lab and observed the activities inside for shorter or longer time from the outside.

**FIGURE 1:**
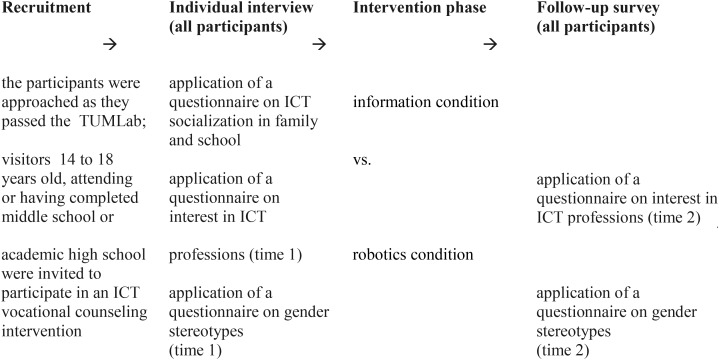
Temporal sequence of the investigation.

The interview started with some open questions on the participants’ museum experience (What was already looked at? What was particularly impressive? What was entertaining and fun?). These questions served as a warming up and were not evaluated for the investigation. Next, we applied several questionnaires (on the participant’s ICT socialization at home and in school, on the self-rated ICT competence etc.). Subsequently, a questionnaire capturing the interest in ICT professions and a short scale on gender stereotypes were applied. Then the vocational counseling intervention (one of two conditions) was carried out and this was followed by a second application of the questionnaire measuring the interest in ICT professions and the gender stereotypes scale (see Figure 1). Overall, the interview plus intervention took two to 3 h per participant. Two alternative methods of informing the participants about the ICT field were tested in the intervention phase of the study (information condition vs. robotics condition). The participants were randomly assigned to these conditions. Each participant was accompanied by a member of the research team who guided her/him through the various stages of the investigation.

### Participants

We selected the participants for the study based on three criteria: age (14–18 years), gender (half male and female participants) and education (ongoing or completed education at middle school or academic high school level). Qualifications at this level in Germany qualify for practical trainings as an IT specialists or a university education in computer science. Young people at this age and level of education know that they need to make career-oriented training choices in the near future. In terms of gender, we have strived for equal group sizes.

### Concept of the Vocational Counseling Interventions

The core content of the two alternatively explored vocational counseling interventions is counseling for the computer science professions. In both interventions the perceived and actual requirements in training and at work were discussed, as well as future fields of specialization and exciting and challenging aspects of the profession (see also Ehrke et al., 2011; Conein and Schwarz, 2015). The need of the participants to make important educational decisions for their lives in the near future was addressed. All contents were presented orally, with a focus on dialogue with the participant.

A total of *N* = 107 participants in the information intervention took part in an ICT career counseling. The following topics were discussed: How to become an IT specialist based on a middle school diploma or an academic high school diploma; authentic, positive statements from computer scientists as well as from employers on the ICT working world, on supposed barriers and on little-known facts from the professional life of IT specialists. Finally, the promotion and earning opportunities and the compatibility of work and family life (flexible working hours, working from a home office) as well as professional, social and communicative skills needed as prerequisites for the profession were explained.

The total of *N* = 27 participants in the robotics intervention joined in a stimulating, knowledgeable, guided tour of the robotics exhibition, which showed the diverse applications of robots in everyday life (luggage robots, household robots, welding robots, milking robots, magnetic tape storage, medical prostheses, etc.). To arouse interest in the computer science professions among the participants, we focused on descriptive explanations based on the exhibits. They were explained in terms of their design, operation, manufacturing and uses, always referring to the tasks of the computer specialists involved. The tour was organized in groups of three people plus a guide from the research team. Due to the public traffic in the exhibition hall it was often very loud there. In the trial week before the study we selected timeslots, to which the hall during the day had little public traffic. In order to facilitate the communication of the contents and the dialogue with the participants, we waited for the implementation until there were few or no persons in the hall. As a result, only a relatively small number of participants were able to take part during the research week. This explains the low number of participants in the robotics condition.

### Development of the Research Scales

All survey instruments used in this study are listed together with the items in Tables 2, 3, 5–9. Computer affinity was recorded with a short *Computer affinity scale* (α = 0.80; *N* = 134). The self-perceived mathematical competence was captured with the short *Scale on math competence* (α = 0.68; *N* = 134). Both items, here slightly reworded and turned positive, are based on the Self-appraised math ability scale Schmader et al. (2004) used with good results. For both dimensions of computer affinity and math competence, longer scales exist that were not used here for reasons of survey economy (see Meelissen and Drent, 2008; Heerwegh et al., 2016; Wille et al., 2018). The three questions on computer literacy and the three questions on Internet literacy were newly developed based on items found in the “ICT Familiarity Questionnaire” [see PISA, (OECD, 2015)]. From these six items the *Maintenance competence scale* (4 items) was obtained by factor analysis (*M* = 15.42, *SD* = 3.41; α = 0.76); the remaining two items indicating the readiness to seek/accept external help and intercorrelate with *r*(134) = 0.54, *p* < 0.001.

There are a number of perceived parental beliefs/parental attitudes scales, all of which have been used in the context of research on gender stereotypes in mathematics (see Wendland and Rheinberg, 2004; Lazarides and Ittel, 2013; Lazarides et al., 2016; Lazarides and Watt, 2017). The *Perceived parental attitudes scale* used in this study consists of six items. It is a short form of the 8-item “Perceived parental support scale” by Vekiri (2010). Vekiri’s scale had a good Cronbach’s alpha of α = 0.84. Due to the reduction made for reasons of survey economy, the homogeneity of our shortened scale has decreased, but is still acceptable (*M* = 20.59, *SD* = 4.13; α = 0.69). Both the 6-items scale and the scales based on each item of this scale were included in the statistical analysis.

The nine statements on the qualities of School computer courses/computer science courses after factor-analytic treatment and reduction by two items led to a new scale named *Qualities of the ICT courses* (*M* = 21.15, *SD* = 5.74; α = 0.79). These items, as well as the items of the *Perceived ICT competence of ICT teachers scale*, were newly developed for the study. The *Perceived ICT competence of ICT teachers scale* in the factor analysis proved to be one-factorial and has a high degree of homogeneity (*M* = 22.60, *SD* = 8.55; α = 0.92).

In the past, questions about interest in ICT have been asked mainly in the context of large, representative surveys (e.g., PISA studies, OECD reports, JIM studies). From the start, STEM research also dealt with this topic. In this study the topic is accessed in a special way. It is not just about the interest in ICT, but about the interest in ICT professions and a career in ICT! The *Interest in ICT professions scale* consists of a total of 17 items. Originally developed for the engineering profession, six items were taken from a study by Kessels and Hannover (2004); two items were taken from a study by McLachlan et al. (2010) on middle school students’ attitudes to ICT. Nine items were newly created, based on discussions with experienced computer scientists, career counselors and computer science professors as well as on research results from ICT gender research. The new questionnaire was first time applied in this study. As expected, the factor analysis confirmed a multidimensional structure of the scale. For the purposes of the present study, however, the scale was only used in its entirety (1st measurement: *M* = 59.11, *SD* = 7.96; α = 0.78; 2nd measurement: *M* = 60.05, *SD* = 8.31; α = 0.81).

Finally, some questions were presented to the participants in order to grasp how deeply gender stereotypes are still anchored in these young participants. The three questions used here have been used more often in gender research, most recently by Papastergiou (2008). The total scale formed from the three items called *Gender_tot_ scale* reached a high Cronbach’s alpha of α = 0.80 on the 1st measurement (*M* = 8.26, *SD* = 3.43) and of α = 0.78 on the 2nd measurement (*M* = 8.37, *SD* = 3.50) in our study.

Statistically, analyses of variance with the factors AGE (14, 16, and 18 years of age), SEX and SCHOOL (middle school vs. academic high school), *t*-tests and Chi^2^-tests, factor analyses, intercorrelations and multiple linear regression analyses were administered to the data.

## Results

### ICT Socialization, User Biography and Self-Assessment of Computer and Internet Competence

To find out what role does ICT socialization (i.e., the individual media use biography) play in the interest in ICT professions, we systematically interviewed the participants. First, we tried to determine how important computers are to their lives. This was followed by another standard question of STEM research, namely the question of the self-esteemed talent for computers. The affinity to computers was examined in the context of two questions (1 = strongly disagree, 5 = strongly agree): “*Do you agree with the statement: The computer was my favorite hobby right from the start!?*” (Hobby 1; *M* = 2.77, *SD* = 1.16) and “*Do you agree with the statement: The computer is my favorite hobby today!?*” (Hobby 2; *M* = 2.84, *SD* = 1.12). In both statements, the participants signaled a barely average interest. They were grouped into the short *Computer affinity scale* (*hobby _tot_*), as already explained in the “Materials and Methods” section. A two-way analysis of variance with the factors SEX × SCHOOL revealed no significant main effects for the *Computer affinity scale*, but a significant interaction, *F*(1,130) = 8,32, *p* = 0.005, $\eta_{p}^{2}$ = 0.06. The highest approval was given by the male academic high school students (*M* = 6.14), the lowest by the female academic high school students (*M* = 5.00).

In the self-assessed math competence, recorded with the statement “*I am good at solving math problems*,” the two-way analysis of variance SEX × SCHOOL (*M* = 3.41, *SD* = 1.19) showed a significant main effect for the factor SEX, *F*(1,131) = 4,12, *p* = 0.044, $\eta_{p}^{2}$ = 0.034, i.e., the girls rated themselves significantly less competent than the boys. The second statement “*It is important to me that I perform well in math problems.*” the two-way analysis of variance SEX × SCHOOL revealed equally high middle-level approval in both genders (*M* = 3.78, *SD* = 1.10), i.e., there were no significant differences for the main effects and the interaction. Since both items intercorrelate with *r*(134) = 0.52, *p* < 0.001, they were grouped into a short *Scale on math competence* (*math_tot_, M* = 7.19, *SD* = 2.00), as already explained in the “Materials and Methods” section.

Asked about the current computer/laptop ownership and the frequency of Internet use, the participants do not differ. However, their user biography differs amazingly in terms of the first PC use and the first Internet use by age and gender. These two biographical questions can be found in the ICT Familiarity Questionnaire for PISA 2015.

Table 1 shows, based on a three-way analysis of variance AGE × SEX × SCHOOL, how the first laptop/PC use and the first Internet use with falling age of the respondents drops significantly: the younger the respondents, the earlier the onset (first computer use: *F*(2, 121) = 3.64, *p* = 0.03, $\eta_{p}^{2}$ = 0.057, first Internet usage: *F*(2, 121) = 5.25, *p* = 0.006, $\eta_{p}^{2}$ = 0.075). Relevant for the ICT socialization in the family is also the statistically significant main effect for the factor SEX at the first PC use. Compared to the boys, the girls in all three age groups first time used a PC with a delay of a whole year, *F*(1, 121) = 6.79, *p* = 0.01, $\eta_{p}^{2}$ = 0.053)! In addition, the two-way analysis of variance shows a significant interaction of the factors SEX and SCHOOL at the first PC use, *F*(1, 121) = 7.34, *p* = 0.008, $\eta_{p}^{2}$ = 0.06). The discrepancy is particularly high for the first use of computers among academic high school students. While the male academic high school students started comparatively early at an average age of 8;5 years, the female academic high school students only started on average 10;0 years!

**Table 1:** Small media use biography (*N* = 134).

First PC use	14 years	16 years	18 years
(in age; months)	8;1	9;3	9;4
	Male		Female
	8;5		9;5
First Internet use	14 years	16 years	18 years
(in age; months)	9;6	10;8	10;9

*There were no gender differences with regard to the first Internet use.*

Related to the results of the self-assessment of the participants’ computer or Internet competence – here the three items each for computer literacy and Internet literacy are involved – there are further differences between the sexes. The statement of being able to run and maintain a computer in all important areas (item1) by running a three-way analysis of variance AGE × SEX × SCHOOL produced a main effect on the gender variable. The girls rated their competence in this area significantly lower. This also applies to the statements 3 and 6, that the participants would in the case of computer problems or Internet problems first try to seek a problem solution on their own. Again, the girls estimate their computer and Internet skills in this area significantly lower than the boys. On the other hand, with regard to the willingness to seek external help in the event of computer or Internet problems, both sexes equally have no problem accessing external help (for the results, see Table 2).

**Table 2:** Computer and Internet competence.

	*M*	*SD*	Three-way analyses of variance
			Age × Sex × School (*N* = 133)
Keeping *the computer* running (e.g., download virus scanner): Is it true that …?			
(1) I can keep my computer running all alone in all important areas!	3.71	1.09	Sex: *F*(1, 122) = 11.57, *p* = 0.000, $\eta_{p}^{2}$ = 0.087, *M*^boys^ = 4.07, *M*^girls^ = 3.28
(2) If I’m unsure or do not know something about the operation of my computer, I have no trouble getting help!	4.31	1.09	n.s.
(3) If I’m unsure or do not know something about the operation of my computer, I try to solve the problem myself!	3.68	1.16	Sex: *F*(1, 122) = 17.30, *p* = 0.000, $\eta_{p}^{2}$ = 0.124, *M*^boys^ = 4.08, *M*^girls^ = 3.20
Using *the Internet* (for example, searching for information from a search engine): Is it true that…?			
(4) I can do everything that matters on the Internet (visit chat rooms, download sound files, maintain the homepage, Skype, use SchülerVZ, post photos/videos, etc.)!	4.45	0.91	n.s.
(5) If I’m unsure or do not know something about the security, individual programs, offers etc., on the Internet, I have no problems getting help!	4.13	1.10	n.s.
(6) If I’m unsure or do not know something about the security, individual programs, offers etc., on the Internet, I try to solve the problem myself!	3.58	1.23	Sex: *F*(1, 122) = 11.73, *p* = 0.001, $\eta_{p}^{2}$ = 0.088, *M*^boys^ = 3.96, *M*^girls^ = 3.13

*n.s.: no significant differences between the groups; scale: 1 = strongly disagree; 5 = strongly agree.*

For the purpose of data reduction, a factor analysis with varimax rotation was performed on the six items. The result was a clear two-factorial structure with a variance resolution of 66.8%. From the four variables loading on the first factor (items 1, 3, 4, 6), the *Maintenance competence scale* was obtained for further statistical analyses (*M* = 15.42, *SD* = 3.41, *N* = 133, α = 0.76). The remaining Items 2 and 5, loading on the second factor (indicating the readiness to seek/accept external help) intercorrelate with *r*(134) = 0.54, *p* < 0.001. As in the case of the individual analyses of the items, the result of the three-way analysis of variance AGE × SEX × SCHOOL of the *Maintenance competence scale* shows a significant main effect for the factor SEX, *F*(1, 212) = 15.93, *p* = 0.000, $\eta_{p}^{2}$ = 0.12. The girls (*M* = 13.95) rated their computer and Internet maintenance skills significantly lower than the boys (*M* = 16.67). On the other hand, for the case of being in need of expert help due to computer or Internet problems (items 2 and 5), all participants will see no or little problems in finding and accepting help (computer problems: *M* = 4.3, *SD* = 1.09, *N* = 134, Internet problems: *M* = 4.1, *SD* = 1.10, *N* = 134).

#### Put Briefly

These first results show that the participants differ significantly, be it in terms of their ICT user biography – the girls were given access much later, especially the academic high school girls – be it in terms of their self-assessment of ICT-related skills, preferences and deficits (i.e., computer affinity, ICT maintenance competence, math competence). In all three areas the male participants rated themselves more competent than the female participants. When it comes to computer affinity, it is noticeable that the female academic high school students even achieve the lowest scores, while the male academic high school students achieve the highest scores.

### Informal Learning Environment “Family”

Trying to have a closer look at family socialization processes, we asked our participants what they believe their parents think about their computer competence and their suitability for an IT job. This was explored with six statements on parental assessment of their skills and abilities on the computer. The first three questions deal with the perceived importance parents attribute to computer literacy among the participants, and the second group of three questions deals with the skills that their parents deem their youth in the ICT field.

While the mean values for items 1, 2, 4 and 5 are between *M* = 3.7 and almost *M* = 4.0 (Table 3), the mean for Item 3 “*My parents encourage me to learn more about computers*” is at a much lower value of *M* = 2.3, i.e., the participants believe their parents are not really interested to influence them to improve their computer skills. However, they still feel supported by them: Statement 5 “*My parents think I’m smart enough to improve my knowledge of computers*” achieves the highest mean, regardless of age, gender and school type.

**Table 3:** Perceived parental attitudes.

	*M*	*SD*
“Is it true that… ?”		
(1) My parents believe that it is important for my future that I’m well versed in computers.	3.69	1.12
(2) My parents think that having computer skills is good for me.	3.77	1.05
(3) My parents encourage me to learn more about computers.	2.31	1.20
(4) My parents are convinced that I’m good at the computer.	3.89	0.93
(5) My parents think I’m smart enough to improve my knowledge of computers.	3.97	0.93
(6) My parents think I’m suitable for an education in computer science.	2.96	1.38

*Scale: 1 = strongly disagree; 5 = strongly agree.*

In statement 4 “*My parents are convinced that I’m good at the computer*,” which most clearly expresses how the participants perceive their parents to assess their computer competence, there is a main effect for the factor SEX, *F*(1,130) = 4.28, *p* = 0.040, $\eta_{p}^{2}$ = 0.032 within the framework of the two-way analysis of variance SEX × SCHOOL. The girls (*M* = 3.70, *SD* = 1.01) compared to the boys (*M* = 4.04, *SD* = 0.84) significantly assume a more critical competence assessment by their parents.

When directly asked, *how their parents would assess their suitability for an education in computer science*, the two-way analysis of variance SEX × SCHOOL finds main effects for both factors as well as a significant interaction: Again there is a main effect for the factor SEX *F*(1, 130) = 10,21, *p* = 0.002, $\eta_{p}^{2}$ = 0.073, i.e., the girls (*M* = 2.56, *SD* = 1.37) expected their parents to assess their suitability for an education in ICT significantly lower than the boys (*M* = 3.30, *SD* = 1.30). The factor SCHOOL, *F*(1, 130) = 9.35, *p* = 0.003, $\eta_{p}^{2}$ = 0.067 is also statistically significant: the middle school students (*M* = 3.31) expect their parents to assess their suitability for an education in computer science significantly higher than the academic high school students (*M* = 2.70). Additional information on this can be found in the interaction SEX × SCHOOL, *F*(1, 130) = 4.24, *p* = 0.041, $\eta_{p}^{2}$ = 0.032: Here the female academic high school students again reach the lowest value of all (*M* = 2.03), i.e., they assume that their parents tend to deny their suitability for an education in computer science!

To further clarify the ICT socialization of the participants, it is also important to find out who has guided them in terms of computer and Internet use as a child. The answers to the question below about the persons in the participants’ social environment (family, school), who were important for teaching her/him ICT skills, provide interesting differences between young women and young men: When asked, “*By whom did you learn the most about computers, the Internet and digital media*?” the Chi^2^ test shows statistically significant differences between female and male respondents (Chi^2^ = 24.85, *df* = 3, *p* = 0.000, Φ_c_ = 0.40)! Table 4 shows that as a matter of course many male participants were introduced to the handling of computers by the father and by the friends, while the female respondents are referred to a very high proportion for the acquisition of computer literacy to teachers.

**Table 4:** Persons in the participants’ social environment (family, school) important for teaching them ICT skills.

	Males	Females
Father	46.6%	29.5%
Mother	–	8.2%
Peers	42.5%	24.6%
Teachers	11.0%	37.7%

#### Put Briefly

These results confirm that the familial ICT socialization of female and male participants in our study has been different. From their parents, the girls expect significantly less compared to the boys that they classify them on the computer as gifted. They themselves doubt their talent for this field. The female academic high school students here again reach the lowest value. Fewer girls than boys were mentored by the father in terms of computer and Internet usage. With the girls, the peers also play only a minor role. All the more, formal education has an important compensatory role to play. In fact, teachers play an important role in ICT socialization of girls. But are they doing justice to this task?

### ICT Socialization in School

Looking at the formal learning environment “school,” there are several construction sites. To find out what the school’s ICT socialization is all about, and what this means for a potential career choice of the students in ICT, the analysis must be in content and personnel. Pedagogically, the legitimate question arises as to how far the school ICT curriculum meets the requirements of a digitized society. Socially, the question arises to what extent the schools are able to compensate for deficits in informal digital socialization of the students (Toomey Zimmermann and Bell, 2012). With regard to central actors, the question arises as to what extent todays teachers are competent to train children and adolescents for a digitized society.

Already in the flashback to the elementary school time the participants’ information point to substantial deficits with equipment and curricula. The difference between boys and girls in the participation in ICT-relevant lessons is striking: 41.1% of the boys, but only 26.2% of the girls (Chi^2^ = 3.26, *df* = 1, *p* = 0.07, Φ_c_ = 0.16), affirmed to have participated in PC courses in primary school. The difference is especially pronounced among academic high school students. Of these, 41.9% of the boys, but only 18.2% of the girls, participated in such courses as early as in primary school (Chi^2^ = 4.58, *df* = 1, *p* = 0.028, Φ_c_ = 0.25).

When asked about the frequency of laptop/notebook classes in school lessons at the middle school or at the academic high school, the participants in our study explained to 73.1% that they had (so far) never participated in such classes. Asked about the use of interactive whiteboards (IWBs) in school lessons (scale: 1 = never to 5 = regularly), 55.2% of respondents said that IWBs were never used. Only 9% of respondents stated that they are used regularly. A significant main effect was found in the two-way analysis of variance SEX × SCHOOL for the factor SCHOOL, *F*(1, 130) = 19.83, *p* = 0.000, $\eta_{p}^{2}$ = 0.132. While in middle schools IWBs are used more often (*M* = 2.64), they are rarely used in academic high schools (*M* = 1.61).

This difference in the use of digital media between middle schools and academic high schools is systematic. Still, as the author herself knows from her many years of cooperation with secondary schools in Germany, the faculty members in academic high schools are more likely to assume that their students “do not need” digital media to support school learning. The question of whether they had already participated in special computer/ICT/computer science courses in their school (middle school or academic high school) was confirmed by 94.8% of all participants, 5.2% said no. The course content was mostly office packages, Internet security and Internet research. 13.8% of middle school students and 28% of academic high school students reported having participated in programming courses (Chi^2^ = 3.87, *df* = 1, *p* = 0.049, Φ_c_ = 0.17). This difference is also statistically significant.

Next, we asked how the participants experienced the computer courses/computer science courses at school. Nine statements on the quality of these courses were presented (later summarized to the *Qualities of the ICT courses scale*). Only the statements 2, 8 and 9 are formulated negatively and include criticism. The responses, analyzed using two-way analysis of variance (SEX × SCHOOL), show that participants appreciated courses that allowed them to run their own projects, courses that related to everyday life, that were dealing with multimedia and web design, and those courses that allowed collaboration and discussions with the peers. For such courses, moderate to medium approval ratings between *M* = 2.9 and *M* = 3.4 were achieved. The female participants more frequently stated that they value collaboration with the peers. The male respondents describe the courses more frequently as boring, dull and repeating content (see Table 5). To prepare further analyses, the nine items were analyzed by factor analysis with varimax rotation. The result was a clear two-factor solution with 50.6% explained variance. The items 1, 3, 4, 5, 6, 7, (-) 8 loaded on the factor *Courses A* “*exciting, everyday-oriented, team-oriented content*” with a homogeneity of α = 0.79. On the factor *Courses B* “*course contents too narrowly designed (programming, technical skills)*” loaded the Items 2 and 9, which intercorrelate slightly with *r*(133) = 0.20, *p* = 0.023. The scale *Courses A*, based on the participants’ very low average values, was identified as suitable for later regression analyses.

**Table 5:** Opinions about the school computer courses/computer science courses.

	*M*	*SD*	Two-way analyses of variance
			Sex × School
“How is it/was it in the school courses (computer courses/computer science courses)? What are/what were your experiences?”			
(1) What I like/always liked in these courses is/was that I can create my own things.	3.08	1.23	n.s.
(2) The topic of programming takes too much space in such courses.	2.30	1.19	n.s.
(3) The tasks in these courses have a connection to my everyday life.	2.86	1.21	n.s.
(4) In such courses, I can use my creativity and imagination.	2.74	1.24	n.s.
(5) I’ve always found topics like multimedia and web design exciting!	3.39	1.19	n.s.
(6) What I like about these courses is that I can work and discuss with my classmates.	3.21	1.23	Sex: *F*(1, 129) = 4.79, *p* = 0.030, $\eta_{p}^{2}$ = 0.036, *M*^boys^ = 3.00, *M*^girls^ = 3.47
(7) The tasks in these courses/in computer science correspond to my interests.	2.71	1.23	n.s.
(8) What we do/have done in the computer/computer science course is usually boring and consists of banal, repetitive tasks.	2.84	1.26	Sex: *F*(1, 129) = 7.48, *p* = 0.007, $\eta_{p}^{2}$ = 0.055, *M*^boys^ = 3.08, *M*^girls^ = 2.55
(9) What we do/have done in the computer/computer science course is often aimed at learning high technical skills, far from issues that are significant and creative.	2.50	1.03	n.s.

*n.s.: no significant differences between the groups; scale: 1 = strongly disagree; 5 = strongly agree.*

As for the ICT competence of the teachers, a total of nine items were presented to the respondents. Overall, the participants rated four negative and four positive statements about the ICT competence of ICT teachers. A critical statement on the ICT equipment of teachers (Item 4) was added. The critical items 1–5 were rated by the respondents with relatively low values between *M* = 2.2 and *M* = 2.4 (1 = strongly disagree to 5 = strongly agree). The four positive statements (items 6–9) had higher values between *M* = 3.3 and *M* = 3.6. Only Item 8 “*My ICT teachers encourage and inspire me*” falls well short of these values with *M* = 2.8 (see Table 6).

**Table 6:** Perceived ICT competence of ICT teachers.

	*M*	*SD*	Two-way analyses of variance
			Sex × School
“My teachers in this field …”			
(1) usually have/had little experience	2.43	1.33	n.s.
(2) are/were technically poorly versed	2.37	1.15	School: *F*(1, 130) = 4.54, *p* = 0.035, $\eta_{p}^{2}$ = 0.034, *M*^MS^ = 2.14, *M*^AHS^ = 2.55
(3) do not know enough or less than me	2.26	1.37	Sex *F*(1, 130) = 22.97, *p* = 0.000, $\eta_{p}^{2}$ = 0.15, *M*^boys^ = 2.74, *M*^girls^ = 1.69
(4) are/were not properly equipped	2.29	1.19	Sex: *F*(1, 130) = 13.30, *p* = 0.000, $\eta_{p}^{2}$ = 0.093, *M*^boys^ = 2.62, *M*^girls^ = 1.90; School: *F*(1, 130) = 8.07, *p* = 0.005, $\eta_{p}^{2}$ = 0.058, *M*^MS^ = 1.97, *M*^AHS^ = 2.54
(5) cannot help	2.19	2.24	School: *F*(1, 130) = 3.76, *p* = 0.055, $\eta_{p}^{2}$ = 0.028, *M*^MS^ = 1.95, *M*^AHS^ = 2.28
(6) are/were very knowledgeable and fully qualified	3.28	1.24	Sex: *F*(1, 130) = 8.46, *p* = 0.004, $\eta_{p}^{2}$ = 0.061, *M*^boys^ = 3.04, *M*^girls^ = 3.57; School: *F*(1, 130) = 10.03, *p* = 0.002, $\eta_{p}^{2}$ = 0.072, *M*^MS^ = 3.62, *M*^AHS^ = 3.03
(7) can/could give much information	3.55	1.05	Sex: *F*(1, 130) = 6,12, *p* = 0.015, $\eta_{p}^{2}$ = 0.045, *M*^boys^ = 3,34, *M*^girls^ = 3,80; School: *F*(1, 130) = 5.95, *p* = 0.016, $\eta_{p}^{2}$ = 0.044, *M*^MS^ = 3.81, MGym = 3.36
(8) encourage and inspire me	2.77	1.23	Sex: *F*(1, 130) = 7.35, *p* = 0.008, $\eta_{p}^{2}$ = 0.062, *M*^boys^ = 2.51, *M*^girls^ = 3.08; School: *F*(1, 130) = 6.06, *p* = 0.015, $\eta_{p}^{2}$ = 0.049, *M*^MS^ = 3.07, *M*^AHS^ = 2.54
(9) know their way around	3.34	1.17	Sex: *F*(1, 130) = 11.81, *p* = 0.001, $\eta_{p}^{2}$ = 0.083, *M*^boys^ = 3.04, *M*^girls^ = 3.70; School: *F*(1, 130) = 4.48, *p* = 0.036, $\eta_{p}^{2}$ = 0.033, *M*^MS^ = 3.59, *M*^AHS^ = 3.16

*n.s.: no significant differences between the groups; scale: 1 = strongly disagree; 5 = strongly agree.*

Overall, participants have a moderately positive opinion on the competence and teaching performance of their ICT teachers. In comparison to the male students, the female students were significantly less critical in the negative statements on the ICT competence of the teachers (Items 3, 4). In the positive statements on competence/teaching behavior (6, 7, 8, 9), they rated their teachers significantly more positive. With regard to the critical statement on the technical competence of the teachers, both sexes agreed with a low positive value of *M* = 2.37. For all items with a significant result on the main factor SCHOOL, the academic high school students were significantly more critical than the middle school students.

For later evaluations, these nine items were also analyzed by factor analysis and the *Perceived ICT competence of ICT teachers scale* was formed. Principal component analysis revealed a one-factorial solution with 62.7% variance (α = 0.92). As with the individual items, the overall scale confirmed the statistically significant results for the two main factors SEX, *F*(1, 130) = 12.30, *p* = 0.001, $\eta_{p}^{2}$ = 0.086, boys = 24.84, girls = 19.93 and SCHOOL, *F*(1, 130) = 8.53, *p* = 0.004, $\eta_{p}^{2}$ = 0.062, *M*^MS^ = 20.22, *M*^AHS^ = 24.42. The girls judge their teachers significantly less critically than the boys. The academic high school students assess them significantly more critical than the middle school students.

#### Put Briefly

It should be noted that the majority of participants in this study experienced less interesting, less inspiring school-based courses on ICT topics. In the classroom, digital media are rarely used, again striking the difference between middle school students (higher use of digital media) and academic high school students. The female participants value those ICT courses significantly higher, which enable cooperation with the peers. They evaluate their ICT teachers significantly more positively than the male participants. This is conclusive, since these teachers are important partners for them in acquiring necessary ICT skills. However, the female participants do not rate the content of the ICT courses more positively! And consistent with the male participants, they evaluated the teachers’ technical skills relatively low.

### Interest in Careers in ICT

In order to find out how interested the participants are in careers in the ICT sector, all participants were asked about their interest in ICT professions, once before the intervention (information condition vs. robotics condition) and once afterward. The 17-item *Interest in ICT professions scale* contains statements that should provide insight into the personal assessment of the costs and benefits of education and professional practice in ICT. As mentioned earlier, the concept of the scale incorporates findings from research, but statements from discussions with ICT experts in research and practice have also been incorporated. Following the survey using the *Interest in ICT professions scale*, a short survey (three items) on gender stereotypes were administered (*Gender_tot_ scale*; see Section “Gender Stereotypes and Their Importance for the Interest in ICT Professions”).

As can be seen from Table 7, there were higher approval ratings for items 1, 3, 5, 7, 8, 9, 12 and 15, which also take up many new developments in the occupational field of ICT. The dimensionality of the *Interest in ICT professions scale* was clarified by means of a factor analysis with varimax rotation: A three-factorial solution was obtained, which showed 46.4% explained variance. The dimension “Classically modern ICT profession” includes the items 1, 3, 5, 7, 8, 9, 14,15, summarizing the profession’s positive characteristics as diverse fields of application, creative possibilities, good pay, good promotion prospects, the potential for change, and the chance to work independently (α = 0.71). The dimension “Flexibility, diversity, social competence” includes items 10, 11, 12, 13,16, the content of dealing with the need for high social competence, the need for many different skills, the good compatibility of work and family (α = 0.67). The dimension “Previous knowledge in mathematics and programming are not a prerequisite” includes items 2, 4, 6, 17 (α = 0.66).

**Table 7:** Interest in ICT professions scale.

	*M*	*SD*
“I find interesting in ICT professions … ”		
(1) … that they offer good opportunities to become active in many areas (e.g., media informatics, environmental informatics, medical informatics, bioinformatics, business informatics).	3.60	1.10
(2) …that mathematics is not a central element of education	3.04	1.26
(3) …that they are creative.	3.60	1.00
(4) …that they are not difficult professions.	2.72	1.13
(5) …that they are respected and well paid.	3.82	0.93
(6) …that previous knowledge in programming is not a prerequisite for education.	3.10	1.12
(7) …that they are occupations that are constantly evolving.	4.19	0.82
(8) …that they are occupations that will always be important for society.	4.06	0.84
(9) …that they are occupations with a variety of tasks.	3.90	0.84
(10) …that they are occupations where you have to adapt to many different people with many different ICT-needs.	3.37	0.93
(11) …that they are occupations in which I can reconcile work and family well.	3.25	0.95
(12) …that I can use many different abilities.	3.61	0.99
(13) …that I am expected to be good with people.	3.25	1.09
(14) …that I also work alone a lot.	3.39	1.14
(15) …that I have good promotion prospects.	3.88	0.85
(16) …that these professions help solve many social problems.	3.50	1.01
(17) … that skills in mathematics are not important for the practice of the profession.	2.87	1.40

*time1 = 1st measurement; total score time1: *M* = 59.11, *SD* = 7.96; scale: 1 = strongly disagree; 5 = strongly agree.*

Differences between groups became only apparent in the dimension “Previous knowledge in mathematics and programming are not a prerequisite.” The three-way analysis of variance AGE × SEX × SCHOOL has a significant effect on the AGE factor, i.e., the optimistic belief that missing math and programming skills are no barrier to the different ICT educational paths decreases with age (*N* = 134, AGE: *M*^14years^ = 12.53, *M*^16years^ = 11.83, *M*^18years^ = 10.81; *F*(2, 122) = 3.67, *p* = 0.028, $\eta_{p}^{2}$ = 0.070). The 17-item comprehensive *Interest in ICT professions scale* was also subjected to a three-way analysis of variance AGE × SEX × SCHOOL. Once again the factor AGE proves to be statistically significant *F*(2, 122) = 6.65, *p* = 0.002, $\eta_{p}^{2}$ = 0.098, i.e., also the interest in ICT professions decreases in general with increasing age (*N* = 134, AGE: M^14years^ = 60.04, M^16years^ = 61.78, M^18years^ = 55.91). It is noteworthy that the factors SEX and SCHOOL have no significant results, i.e., that the interviewed girls and boys as well as the students/graduates of the two school types do not differ in their interest in the ICT professions. After all, it is encouraging and should not be overlooked that this interest reaches on average medium high values of 59 and 60 out of 85 possible points in the first and second measurements!

The three subscales measuring different dimensions of the interest in ICT professions have acceptable homogeneity values for research purposes. As a criterion for regression analysis, however, the 17-item comprehensive *Interest in ICT professions scale* (*M* = 59.11, *SD* = 7.96, α = 0.78) is used. On second use after intervention, the scale proved to be stable (*M* = 60.5, *SD* = 8.31, α = 0.81). In terms of statistics and content, the *Interest in ICT professions scale* is therefore well suited to clarify which aspects of the media biography of the participants best predicts this ICT interest on a correlative basis.

### Examination of Hypotheses by Multiple Linear Regression Analyses

In order to further determine the importance of personal media competence, the informal learning environment “family” as well as the formal learning environment “school” for the interest in careers in the ICT sector, the previously developed indexes and scales were subjected to an inspection of the intercorrelations. It turned out that only few scales are suitable as predictors for the criterion scale *Interest in ICT professions*.

The scales, that have statistical significant correlations with the scale *Interest in ICT professions*, are: the *Computer affinity scale (hobby_tot_*), *r*(134) = 0.28, *p* = 0.001, the *Maintenance competence scale, r*(133) = 0.42, *p* = 0.000, which measures the computer and Internet competence skills, the *Perceived parental attitudes scale, Item 4 (Parents4 scale), r*(134) = 0.39, *p* = 0.000, which captures how the participants perceive their parents judgment on their computer competence, and the scale *Quality of the ICT courses, r*(133) = 0.34, *p* = 0.000, which summarizes the participants’ evaluation of the school computer or computer science courses. The scales on the self-assessed mathematics competence, on the perceived ICT competence of the ICT teachers, and surprisingly also the gender stereotypes’ scale (to be explained in the next section) do not show statistically significant correlations to the criterion.

Next, standard and stepwise versions of linear multiple regression analyses were carried out and led to identical results. Here the results of the standard versions are reported. The linear multiple regression analysis was performed for the whole group (*N* = 134) and also separately for the female and male participants. It showed that the scale *CoursesA* with its previously documented very low participants’ ratings, calculated for the whole group did not contribute significantly to the regression result. In the subsequent analyses of the subgroups, it was completely eliminated from the final regression equation. In a next step, only the scales *Computer affinity/hobby_tot_, Maintenance competence* and *Parents4* were included in the regression analysis. The result of the multiple linear regression analysis indicates that there is a collective significant effect between the three predictors. For the overall group of participants, the prediction of the *Interest in ICT professions* (criterion) with these three predictors yields a good variance explanation of *27%* (*R*^2^ = 0.27, *R*^2^adj = 0.25, *F*(3, 129) = 15.62, *p* = 0.000). It turns out that the three predictors *Maintenance competence* (*ß* = 0.27, *p* = 0.0024), *Parents4* (*ß* = 0.25, *p* = 0.0037) and *Computer affinity/ hobby_tot_* (*ß* = 0.21, *p* = 0.0059) almost equally contribute to the prediction of the criterion.

When performing separate regression analyses for the sexes, the young womens’ interest in ICT professions is better predicted than that of the young men by the three predictors: For the young women, the three predictors yield a high explained variance of *45%* (*R*^2^ = 0.45, *R*^2^adj = 0.42, *F*(3, 57) = 15.58, *p* = 0.000 with the scales *Maintenance competence* (*ß* = 0.37, *p* = 0.0036), *Computer affinity /hobby_tot_* (*ß* = 0.29, *p* = 0.0074) and *Parents4* (*ß* = 0.24, *p* = 0.042). Thus, as hypothesis 1 assumes, these three variables explain the interest of young women in computer science careers. Not only the self-assessment of one’s own computer and Internet competence, which is based on personal learning history, plays a decisive role here. Rather, the enthusiasm for everything that has to do with computers plays an important role. Added to this, the belief of the parents in the daughter’s computer science talent is a very important variable. All three variables predict the interest of female participants in computer science professions.

For the young men, the predictors yield a variance explanation of *20%* (*R*^2^ = 0.20, *R*^2^adj = 0.16, *F*(3, 68) = 5.60, *p* = 0.002), whereby their interest in careers in IT can be significantly predicted with the two predictors *Maintenance competence* (*ß* = 0.26, *p* = 0.02) and *Parents4* (*ß* = 0.25, *p* = 0.03) only. The predictor *Computer affinity/hobby_tot_* is not significant in the standard version of the multiple linear regression analysis, and in the stepwise version this predictor is omitted in step 2 of the analysis. This is an interesting result, calling for further psychological clarification of the background of the male participants’ interest in ICT professions in future research.

#### Put Briefly

The results of the regression analyses and the preceding statistical comparisons lead to the conclusion that the participants’ self-assessed computer and Internet literacy and the parents’ belief in their childrens’ computer science talent decisively determines their interest in IT professions. For the female participants, their enthusiasm for everything that has to do with computers plays an important role. All skills and motivational variables relevant to develop an interest in a career in the ICT sector are presently based on support measures that are predominantly offered or not offered in the private sphere. The results confirm hypotheses 1 and 2.

### Effect of Short-Term Interventions on the Interest in ICT Professions

We asked ourselves: Can a short-term vocational counseling intervention – here called information intervention vs. robotics intervention – increase the participants’ interest in ICT professions? Which attitude patterns are activated by these two interventions? After having completed either the information intervention or the robotics intervention, the participants for the second time answered the *Interest in ICT professions scale* as well as, also again, the three items of the gender stereotype scale. In paired *t*-tests, the total group of participants as well as the gender-separated subgroups were analyzed separately for the two interventions. We looked for significant changes, item per item and for the scale as a whole.

Calculated separately for the intervention groups, the participants in the information condition (*N* = 107) showed a slightly higher, statistically significant, interest in ICT occupations (*M* = 11.59 to *M* = 12.57, *t* = –3.57, *df* = 106, *p* < 0.001, *d* = 0.33), i.e., the positive rating of ICT occupations was increased by the intervention. For the small group of participants of the robotics intervention, the values decreased slightly after the intervention, but not statistically significant (*M*^time1^ = 12.22, *M*^time2^ = 11.89). This result should be treated with caution given the small sample size of this intervention. Nevertheless revealing, if only purely exploratory, is the pattern of attitude changes to ICT occupations in both interventions.

The results show that the male participants of the information condition have benefited from the enlightening content. They prove with significant higher values in the second measurement that they can change their attitudes in the short term. The intervention increased their interest in key areas. After the intervention, they were more convinced that ICT occupations are not insurmountably difficult, that they do not need any programming background as a prerequisite for education and that the ability to deal with people is necessary. However, less than before, they were convinced that computer science degrees lead to many different jobs. With the girls of the information condition, no statistically significant changes caused by the intervention can be detected.

The boys of the robotics condition rated the variety of own abilities that can be applied after the intervention higher than before, while the girls rated the role of social skills as less important after the intervention than before. All in all, although only a small sample was recorded in the case of the robotics intervention, the constructive processing of new positive information about the training and the profession of IT specialists seems to be more difficult for the female participants in both intervention conditions than for the male participants. There is even a slight tendency for the girls to call in question previously identified positive professional characteristics (see Table 8, robotics intervention).

**Table 8:** Attitude change after interventions^a^.

	*M*^time1^ *M*^time2^	Paired *t*-test results
“I find interesting in ICT professions…”		
1. Information condition: males (*N* = 57)		
(4) that they are not difficult professions. ↑	*M*^time1^ = 2.68, *M*^time2^ = 3.18	*t* = –3.77, *df* = 56, *p* < 0.001, *d* = 0.45
(6) that previous knowledge in programming is not a requirement for education. ↑	*M*^time1^ = 3.05, *M*^time2^ = 3.39	*t* = –2.10, *df* = 56, *p* < 0.05, *d* = 0.27
(9) that they are occupations with a variety of tasks. ↓	*M*^time1^ = 4.04, *M*^time2^ = 3.68	*t* = 3.35, *df* = 56, *p* < 0.01, *d* = 0.41
(13) …that I am expected to be good with people. ↑	*M*^time1^ = 3.04, *M*^time2^ = 3.44	*t* = –3.30, *df* = 56, *p* < 0.01, *d* = 0.40
2. Information condition: females (*N* = 50)		no statistically significant attitude changes
3. Robotics condition: males (*N* = 16)		
(12) that I can use many different abilities. ↑	*M*^time1^ = 3.44, *M*^time2^ = 3.94	*t* = –2.24, *df* = 15, *p* < 0.05, *d* = 0.50
4. Robotics condition: females (*N* = 11)		
(13) that I am expected to be good with people. ↓	*M*^time1^ = 3.36, *M*^time2^ = 3.00	*t* = 2.39, *df* = 10, *p* < 0.05, *d* = 0.60

*^*a*^Statistically significant change items only; *N*^*Inf*^ = 107 (participants information condition), *N*^*Rob*^ = 27 (participants robotics condition); scale: 1 = strongly disagree; 5 = strongly agree; The arrows (↑↓) signal the direction of change.*

#### Put Briefly

Although both interventions led to a change in individual attitude dimensions, only the information intervention increased the interest in the ICT sector statistically significantly. Informative and targeted advisory interventions can thus change the interest in ICT professions in the short term as stated in Hypothesis 3. But this result should be treated with caution, because primarily the male participants benefited from this intervention. They constructively processed the new information on the ICT domain. Also, the long-term after-effects and the sustainability of our interventions could not be recorded in the absence of follow-up opportunities. Nevertheless, the results of these short-term interventions give important indications that we have to decide carefully about what content is taught to whom on the subject of information and communication technologies.

### Gender Stereotypes and Their Importance for the Interest in ICT Professions

The question how gender stereotypes influence the interest in ICT training is a focus of current studies (Galdi et al., 2017; Ihme and Senkbeil, 2017; Förtsch et al., 2018; Lloyd et al., 2018). In this study, the extent to which common gender stereotypes are still effective was assessed with three questions before and after the intervention. The three items and the overall scale formed from these items (Gender_tot_/time 1, α = 0.80; follow-up: Gender_tot_/time2, α = 0.78) were tested with a three-way analysis of variance, using the already known factors SEX and SCHOOL and the new variable Learned_F(learned from the father), which records whether the participants have learned their knowledge about computers and digital media primarily from the father or from other people. After all, almost half of the male participants and almost a third of the female participants have their computer knowledge acquired from the father.

The results of the first variance analysis listed in Table 9 show that the boys who acquired their computer knowledge primarily from the father quite strongly internalized the gender stereotype “*Computer science suits men better than women.*” By contrast, the girls, who learned the most about computers, the Internet and digital media by their father, are the least convinced of this gender stereotype. Also, all female participants in the study are significantly less convinced than the male participants of the statement, “*By nature men are more IT/ICT oriented than women.*” These are signals for change.

**Table 9:** Gender Stereotypes in ICT.

	*M*	*SD*	Three-ways analyses of variance
			Sex × School ×Learned_F(time1)
“My opinion on ICT professions is … ”			
(1) Computer science suits men better than women.	2.68	1.44	School: *F*(1,126) = 3.06, *p* = 0.039, $\eta_{p}^{2}$ = 0.024, *M*^MS^ = 2.36, *M*^AHS^ = 2.92 Sex × Learned_F: *F*(1, 126) = 5.72, *p* = 0.018, $\eta_{p}^{2}$ = 0.043; boys who have learned from the father achieved the highest value (*M* = 3.18), girls who have learned from the father had the lowest value (*M* = 2.17)
(2) Men have more chances to succeed in the IT/ICT sector than women.	2.84	1.36	School: *F*(1,126) = 5.58, *p* = 0.020, $\eta_{p}^{2}$ = 0.042, *M*^MS^ = 2.45, *M*^AHS^ = 3.14
(3) By nature men are more IT/ICT oriented than women.	2.84	1.34	Sex: *F*(1,126) = 4.68, *p* = 0.032, $\eta_{p}^{2}$ = 0.036, boys = 3.07, girls = 2.57
GENDER_tot_ Scale/time2	8.37	3.50	Sex: *F*(1,126) = 5.56, *p* = 0.020, $\eta_{p}^{2}$ = 0.042, boys = 8.90, girls = 7.72 School: *F*(1,126) = 4.36, *p* = 0.039, $\eta_{p}^{2}$ = 0.033, *M*^MS^ = 7.41, *M*^AHS^ = 9.09 Sex × Learned_F: *F*(1, 126) = 3.65, *p* = 0.058, $\eta_{p}^{2}$ = 0.027; boys who have learned from the father achieved the highest value (*M* = 9.59), girls who learned from the father achieved the lowest value (*M* = 7.17)

*M^*MS*^ = means of middle school students, *M*^*AHS*^ = means of academic high school students; Gender_*tot*_ Scale = total scale on Gender Stereotypes in ICT (time1 = 1st measurement; α = 0.80, time2 = 2nd measurement; α = 0.78); scale: 1= strongly disagree; 5= strongly agree.*

An unexpected result is: The gender scales in this study, tested for the three single items and the overall *Gender_tot_ scale*, show no significant correlations (values < *r* = 0.10) with the *Interest in ICT professions scale*. Also, with the other scales, they are just as low or uncorrelated!

However, as already seen in previous results, academic high school students are more “conservative” than middle school students: in our sample girls and boys who attend academic high school or graduated from academic high school have internalized common gender stereotypes significantly stronger. The effects of the two vocational counseling interventions on the gender stereotypes was analyzed by a paired *t*-test for the measurement times before and after the intervention, each separated by intervention and gender, at the three single items and the *Gender_tot_ scale*. With one exception, gender attitudes have not changed after the interventions. The exception concerns the group of male participants in the information condition. They agreed significantly less on the statement “*Computer science suits men better than women*” after the intervention (*M*^time1^ = 2.84, *M*^time2^ = 2.60 (*t* = 2.03, *p* = 0.047, *d* = 0.26).

It should be noted with these results that the approval of the gender stereotypes, be it on the three single items or on the GENDER_tot_ scale, is in the lower middle range for all participants. Addressed directly to common gender stereotypes, the female and male participants in the study show no “strong” reactions. The description of ICT professions as a typical “male” domain did not meet with the female participants’ approval.

#### Put Briefly

Overall, from these previously described results, it can be concluded that the implicit core of Hypothesis 4 “Interest in ICT is influenced by gender stereotypes” is consistent. The ICT socialization conditions in informal (family) and formal (school) education are gendered and designed to hinder gender-equitable access to ICT education and access to ICT careers for girls and young women. Frequently important players (fathers, teachers) in the lives of girls contribute to this situation. However, interest in the ICT sector guided by personal convictions is also present among girls. It is noteworthy that the early introduction to computers by the father reinforces gender stereotypical attitudes in the boys, while significantly attenuates these attitudes in the case of father-trained girls.

The male participants in this study approach the ICT sector with much more self-confidence. In all ICT competency dimensions, they achieve average to higher scores in their self-assessment. They have internalized gender stereotypes, albeit on the whole rather weak. They constructively develop this masculine domain and turn out to be versatile when it comes to processing new information about the ICT sector. Both developments confirm hypothesis 4, but there are also signs of change. In terms of their interest in ICT professions, the surveyed young women and young men, as shown before, do not differ! *Both sexes* show a medium-high interest in this professional field.

## Discussion

All four hypotheses presented in the introduction could be confirmed. The learning history of the participants shows that still today it requires a lot of motivation and initiative on the part of young women, but also of young men, in order to be confident in and strive for future professional activities in the ICT domain. By focusing the investigation on a selected group of well researched variables that might influence young peoples’ career choice in ICT it becomes clear that the time is right to move forward and to pursue this topic strictly solution-oriented (see Meelissen and Drent, 2008; Miliszewska and Sztendur, 2010; Murphy et al., 2010; Nagy et al., 2010; Jagacinski, 2013; Heerwegh et al., 2016; Lazarides et al., 2016; Sáinz et al., 2016; Ertl et al., 2017; Ihsen, 2017; Förtsch et al., 2018).

The results of the regression analyses and the preceding statistical comparisons confirm *hypothesis 1* that the dimension of self-assessed computer and Internet literacy decisively determines the interest of participants in ICT professions. This is especially true for young women interested in a career in IT. It also applies to the family environment and the support measures provided by families. In the family context, the girls experience significantly less support in learning ICT skills, both factually and psychologically. Thus, the individual variable “computer affinity” plays an important role for their interest in ICT professions. The late ICT socialization, the low level of guidance in the private sphere and the reliance on compensatory educational offers by the schools put them at a disadvantage. These facts must also be seen in the context of the much lower self-confidence of female participants assessing their own knowledge and skills in the field of ICT. Developing high computer affinity under such conditions is difficult and only achieved by a fraction of them.

*Hypothesis 2* is also confirmed in numerous results of statistical group comparisons and in the results of the regression analyses: The educational offerings of the schools, which are hardly anchored in the school curriculum, are too rare, too unsystematic and qualitatively insufficient to provide solid ICT knowledge in order to stimulate an interest in ICT professions. The milder judgment of female participants on the competence of ICT teachers and the quality of their courses does not mean that the teachers’ efforts have led to a compensatory achievement, – it signals gratitude. According to the regression analyses, these school offers have no lasting influence on the young womens’ interest in ICT professions. Overall, these results show that, considering the low quality of ICT course content and the participants’ low ratings of their ICT teachers’ ICT competence, todays schools’ influence is too weak to motivate male and female students to seek an education and a career in the ITC sector. The task of compensating socialization deficits among girls and young women can’t be fulfilled by the schools.

Although both vocational counseling interventions led to a change in individual attitude dimensions, only the information intervention increased the interest in the ICT sector statistically significantly. Informative and targeted advisory interventions can thus change the interest in ICT professions in the short term as stated in *hypothesis 3*. The fact is: in this case, in this investigation, the male participants primarily benefited from this intervention.

It is noteworthy that the early introduction to computers by the father reinforces gender stereotypical attitudes in the boys, while significantly attenuates these attitudes in the case of father-trained girls. It is also noteworthy that *both sexes* show a medium-high interest in this professional field. From the previously described results on gender stereotypes, it can be concluded that the implicit core of *hypothesis 4* “Interest in ICT is influenced by gender stereotypes” is consistent. The ICT socialization conditions in informal (family) and formal (school) education are gendered and designed to hinder gender-equitable access to ICT education and access to ICT careers for girls and young women. Important players (fathers, teachers) in the lives of girls still contribute to this situation. At the same time, they are, at least temporarily, a key to solving these problems.

### The Interest in ICT Professions and Gender Stereotypes

The *Interest in ICT professions scale* addresses important aspects of the requirements for a career in the ICT industry. Due to the scale’s dimensionality, it is advisable to further develop it based on the structures found. Both sexes showed no statistically significant differences before and after the interventions. Both genders consistently show a medium-high interest in ICT careers. Intervention-related changes indicate that it is important which content is passed on to young people at this age in the context of vocational counseling. For this it is important to clarify what the psychological processes involved in career choice are all about, especially, how professional interests develop. A new approach to the development of interests (in general) could be helpful: O’Keefe et al. (2018) recently published the results of several experiments on peoples’ implicit theories of the development of personal interests. The authors distinguish two groups of implicit/commonsense theories they have found in their investigations. The first group includes implicit theories that are relatively fixed (“fixed theory”), i.e., the person assumes that a once found interest/passion must be pursued with the highest motivation (excluding other interests); the resulting problems are often underestimated. The second group includes individuals’ implicit theories based on the assumption that personal interests are developing (“growth theory”). O’Keefe and colleagues could show in a series of experiments that people with a fixed theory more quickly lose interest in the pursued passion if problems arise compared to people with an implicit growth theory of interest development (O’Keefe et al., 2018).

A research approach that incorporates these findings and relates them to career choice processes in young women could provide important information. As our research results show one key ingredient in the young women for a positive attitude toward the ICT domain is a long-standing enthusiasm for computers/computer science. This distinguishes them from the male participants in the study, who approach the topic in a more pragmatic way. In particular, the development of effective vocational counseling intervention could benefit from this new research on interest development.

Clayton et al. (2009, p. 153) in a research review on the role of gender stereotypes in ICT came to the conclusion that gender stereotypes “provide misleading ideas about ICT as a career discouraging both girls and boys.” In a more recent study published by Banchefsky and Park (2018, p. 1), the authors researching gender-science stereotypes in male-dominated academic disciplines could prove that their female participants were “significantly less likely to endorse the gender-science stereotype.” In the introduction to this article the question was asked “*Can the interest in ICT counteract the masculine image of computer science?*” Based on the lessons learned from their projects, the two research teams of Lasen (2010) and Sáinz et al. (2016) agreed that a new understanding of the tasks and professions in the ICT business already show a positive impact on the interest of girls and young women in ICT careers. Not only statistically, also noteworthy in content is the missing correlation of the reliably measuring gender stereotypes scale (Gender_tot_) with the scale “Interest in ICT professions.” As in the project of Papastergiou (2008) both sexes’ approval of the gender stereotypes is in the lower middle range. What role do these gender stereotypes actually play today? These results probably indicate a step in the right direction: Apparently, the female respondents in our study are interested in the ICT area regardless of how they assess this domain, which they nevertheless perceive as gendered. They realistically value the ICT work environment and the problems that await them there, as triggered by domain masculinity. In any case, they separate between their interest in ICT professions and the perceived masculinity of the ICT domain.

### Limitations

As the results of the survey show, the participants in the study were motivated to deal with the subject of ICT professions. Their interest in this professional field was well above average. This can be interpreted as a limitation of the relevance of the test results due to the selection of the participants. After all, the *Deutsches Museum* in Munich is a technology museum. By contrast, it can be contended that the museum is visited by families, groups of students and tourists from all over Germany and from abroad, usually as a half or full day trip. It may also be argued that the study identifies shortcomings in the education system in Germany that apply only to a specific region. However, anchoring the study locally in the *Deutsches Museum* had the advantage that students from all over Germany were interviewed. That the study sample is lacking in representativeness is already indicated by the sample size.

Another limitation concerns the vocational counseling intervention part of the study: In the absence of follow-up opportunities, we were unable to detect whether our interventions had long-term effects and how they might have turned out. Our interventions served as a test. It was proven that the information provided during the interventions was processed differently by the female and male participants in the study and, to a limited extent, produced short-term effects. More research in this area is needed to follow up on the effects of the interventions over a longer period of time (e.g., several months).

In general, it is recommended that interventions at the current level of gender and STEM research are used more frequently. They lead to revealing test phases, in this study as well as in the actual study by Wheeler and Blanchard (2019). Beyond the hoped-for effects (e.g., Boaler et al., 2018), interventions provide information about the (possibly changing) validity of already investigated important factors that influence education and professional choice in STEM subjects (Wang et al., 2013). Generational, cultural and age-related topics and preferences come into play too (Han, 2019; Wheeler and Blanchard, 2019). Also, interventions offer the opportunity to reveal one-sidedness in research. In today’s view, the assessment of Hsieh et al. (2019) is to be agreed that it should be avoided to study the STEM disciplines as if they were a single subject. In fact, the later professions, which build on the various training paths in the STEM disciplines, have very different profiles.

### Surprising Results

The study also showed some surprising results: as the survey involved interviewing participants with two different (desired or already achieved) school qualifications, it seemed appropriate to look at these groups separately. Surprisingly, it turned out that the female academic high school students had the biggest gap in their ICT socialization/training. As far as these students are concerned, it can be assumed that the socialization disadvantages identified in this study affect them strongly. Apparently, they are still educated and trained according to an outdated “humanistic” educational ideal. In this study, they are the ones whose first use of computers took place very late; they critically consider their mathematical knowledge and their ICT-maintenance competence. They give the lowest ratings in the parental assessment of their computer skills. They are also the most critical of parents’ attitude to their suitability for an ICT profession. While their male classmates have the highest computer affinity despite the low level of computer science training at school, their average computer affinity is lowest. So it is not surprising that the number of female computer science students is growing very slowly. But there is a ray of hope: measures of informal ICT education (family) – currently mostly mediated by the father – seem to be quite effective in establishing computer skills and computer affinity in girls. In terms of gender stereotypes, an increased involvement of the fathers can have an immunizing effect.

Although the results of this small study can only claim a limited scope of validity, they nevertheless offer many starting points for gender-sensitive access to ICT education at all ages (from primary school pupils to high school graduates). As early as possible, digital media have to be firmly anchored as educational media in the consciousness of the younger generation. Something has to change in the informal, family driven as well as the formal education at school. For years, the universities have enthusiastically organized “Boy’s Days” and “Girl’s Days” to encourage children to become interested in STEM fields at an early age. In fact, it requires “Parents’ Days” for parents of children of all ages! The schools, in this study especially the middle schools and the academic high schools have deficits in their curricula and in relation to the ICT competence of the teaching staff. These deficits must be reduced rapidly in the coming years. Digital education should start early and be firmly anchored in the primary school curriculum in order to counterbalance early deficits in access and knowledge of information and communication technologies. But even in adolescence, the ICT competence of students can still be successfully promoted if the contents are clearly anchored and chosen wisely. Much can be achieved on every school-based training level, if one offers a high-quality, lasting and interesting ICT curriculum in the schools and at the same time does not forget to additionally get the parents on board.

Parents, fathers as well as mothers, play an important role in communicating ICT-relevant skills, and above all in educational digital agenda setting. Almost equally important is the emotional support of parents for the children and adolescents interested in ICT education. The fact that fathers are currently better able to provide gender-appropriate ICT guidance for girls is probably a time-bound result that can be overtaken by future social developments. But that is why it is no less relevant! This is a research result that we share with the authors Gunderson et al. (2012a,b), Galdi et al. (2017), Ihme and Senkbeil (2017), and Lloyd et al. (2018).

Today’s students and graduates have a new need for gendered “normality.” They learn self-directed, and with the teaching of ICT skills, the peers play an important role in both sexes. Gender stereotypes have statistically little or no relevance to the other ICT-relevant variables in this study, although they are still present in the gendered training biographies of the girls and boys in this study. After all, according to our results, gender-appropriate, target group-oriented, high-quality school education was not available to our male participants too. However, they approach the topic with a higher self-esteem. An important step toward easing and objectifying this imbalance would be to make the subject of computer science in Germany a standard subject in the curriculum, as is the case with the subjects of physics, chemistry and biology.

## Ethics Statement

The study was approved by the *ad hoc* Ethics Committee of Faculty II of the University of Siegen. The commission follows the ethics rules and the rules of procedure for local ethics committees of the German Psychological Society. In the study, 14–18-year-old participants were approached in the context of their visit to the Deutsches Museum in Munich, Germany, to participate and were informed about the purpose and content of the survey. The participants came to the museum with their families. Oral informed consent was obtained from the parents of the participants; those participants who were of age decided on their own to participate. The consent procedure was approved by the ethics committee.

## Author Contributions

The author confirms being the sole contributor of this work and has approved it for publication.

## Conflict of Interest Statement

The author declares that the research was conducted in the absence of any commercial or financial relationships that could be construed as a potential conflict of interest.
